# Cytoplasmic Pin1 expression is increased in human cutaneous melanoma and predicts poor prognosis

**DOI:** 10.1038/s41598-018-34906-6

**Published:** 2018-11-15

**Authors:** Xin Chen, Xiaosong Liu, Bin Deng, Magdalena Martinka, Youwen Zhou, Xiaopeng Lan, Yabin Cheng

**Affiliations:** 1 Institute for laboratory Medicine, Fuzhou General Hospital, PLA, Fuzhou, Fujian China; 2 Department of General Dentistry, The 174th Hospital of Chinese PLA (Chenggong Hospital affiliated to Medical School of Xiamen University), Xiamen, Fujian China; 3 School of Pharmaceutical Sciences, Fujian Provincial Key Laboratory of Innovative Drug Target Research and Center for Stress Signaling Networks, Xiamen University, Xiamen, Fujian China; 4 Department of Anesthesiology, Xiang’an Hospital of Xiamen University, Fujian, China; 5 Department of Pathology, Vancouver General Hospital, Vancouver, BC Canada; 6 Department of Dermatology and Skin Science, Vancouver Coastal Health Research Institute, University of British Columbia, Vancouver, BC Canada

## Abstract

The prolyl isomerase Pin1 is widely over-expressed or over-activated in cancers and promotes tumorigenesis. The authors investigated the expression level of Pin1 and analyzed the prognostic value of Pin1 expression using a large-scale melanoma tissue microarray study. Two independent sets of tissue microarrays were employed, including 114 melanoma cases in the discovery set and 424 in the validation set (538 cases in total), 32 normal nevi and 86 dysplastic nevi 118 cases of nevi. The subcellular Pin1 expression in different stages of melanocytic lesions and its prognostic significance were studied. High expression (IRS 0–8) of cytoplasmic Pin1 was observed in 3.13%, 8.33%, 16.49% and 22.76% of the biopsies in normal nevi, dysplastic nevi, primary melanoma and metastatic melanoma, respectively. Significant differences for cytoplasmic Pin1 staining were observed between normal nevi and metastatic melanoma (*P* = 0.011, χ^2^ test), between dysplastic nevi and primary melanoma (*P* = 0.046, χ^2^ test) and between dysplastic nevi and metastatic melanoma (*P* = 0.016, χ^2^ test). Kaplan-Meier survival analysis showed that increased cytoplasmic Pin1 expression was associated with a worse 5-year melanoma-specific survival of melanoma (*P* < 0.001) and metastatic melanoma patients (*P* = 0.004). Multivariate Cox regression analysis showed that cytoplasmic Pin1 expression is an independent prognostic factor in melanoma. Our data indicate that cytoplasmic Pin1 plays an important role in melanoma pathogenesis and progression, and serve as a potential prognostic marker for melanoma.

## Introduction

Pin1(peptidyl-prolyl cis-trans isomerase NIMA-interacting 1) is a unique cis-trans isomerase (PPlase) specifically catalyzing isomerization of phospho-serine/threonine-proline motifs and thus inducing protein conformational changes^1^. At the N-terminus, Pin1 has a WW domain that recognizes phosphopeptides, while at the C-terminus it contains a PPIase domain that has catalytic activity. As a consequence of isomerization by Pin1, the stability, subcellular localization and post-translational modifications of the substrates are profoundly affected^2^. Pin1-mediated proline-directed protein phosphorylation is essential in many cellular processes, such as cell proliferation and transformation^3^. Normally, Pin1 is tightly regulated and its deregulation causes multiple diseases, including cancer^4^.

An early study of 60 different human tumor types showed increased Pin1 expression in 38 of these tumors, including prostate, breast, lung, ovary, cervical tumors, and melanoma^5^. Follow-up studies showed that Pin1 expression is associated with poor cancer prognosis^6,7^. Functional studies revealed that Pin1 over-expression leads to abnormal cell cycle regulation and chromosome instability^8^. Pin1 activates more than 40 oncogenes and inactivates approximate 20 tumor suppressors^9^. Although Pin1 is an essential factor for cancer cell growth, it is dispensable for normal cell growth. Pin1-^−/−^ mice are viable, develop normally, and show no obvious defects at young ages^10^. Moreover, Pin1^−/−^ mice are highly resistant to oncogenesis induced by over-expression of oncogenes such as *HER2* and *HRAS* or after inactivation of the tumor suppressor gene *TP53*^11–14^. In addition, Pin1 inhibition sensitizes breast cancer cells to multiple chemo-therapies and targeted drugs^15–18^. Taken together, these results strongly suggest a pro-oncogenic role of Pin1 and provide a sound rationale for developing specific Pin1 inhibitors for treating cancer.

Malignant melanoma, arising from uncontrolled proliferation of melanocytes, is an aggressive form of skin cancer with a rapidly increasing incidence worldwide^19^. Once metastasis occurs, melanoma can hardly be treated; only 14% of metastatic melanoma patients survive for 5 years^20^. Recent development of the targeted inhibitors of specific MAP kinase and the immune checkpoint monoclonal antibodies has been notably improved the treatment of metastatic melanoma^21^. Both therapies have shown survival benefits for patients with metastasis, albeit with limitations^22^.

Based on the widely accepted oncogenic role of Pin1 in cancer, we hypothesized that Pin1 would have profound impact on melanoma pathogenesis and progression. To investigate the role of Pin1 in melanoma progression, we checked Pin1 expression in different stages of melanocytic lesions using tissue microarray (TMA) and immunohistochemistry. Our findings provide strong evidence that cytoplasmic Pin1 expression is a prognostic marker and a promising therapeutic target in melanoma.

## Results

### Clinicopathologic Features of TMAs

**Figure 1:**
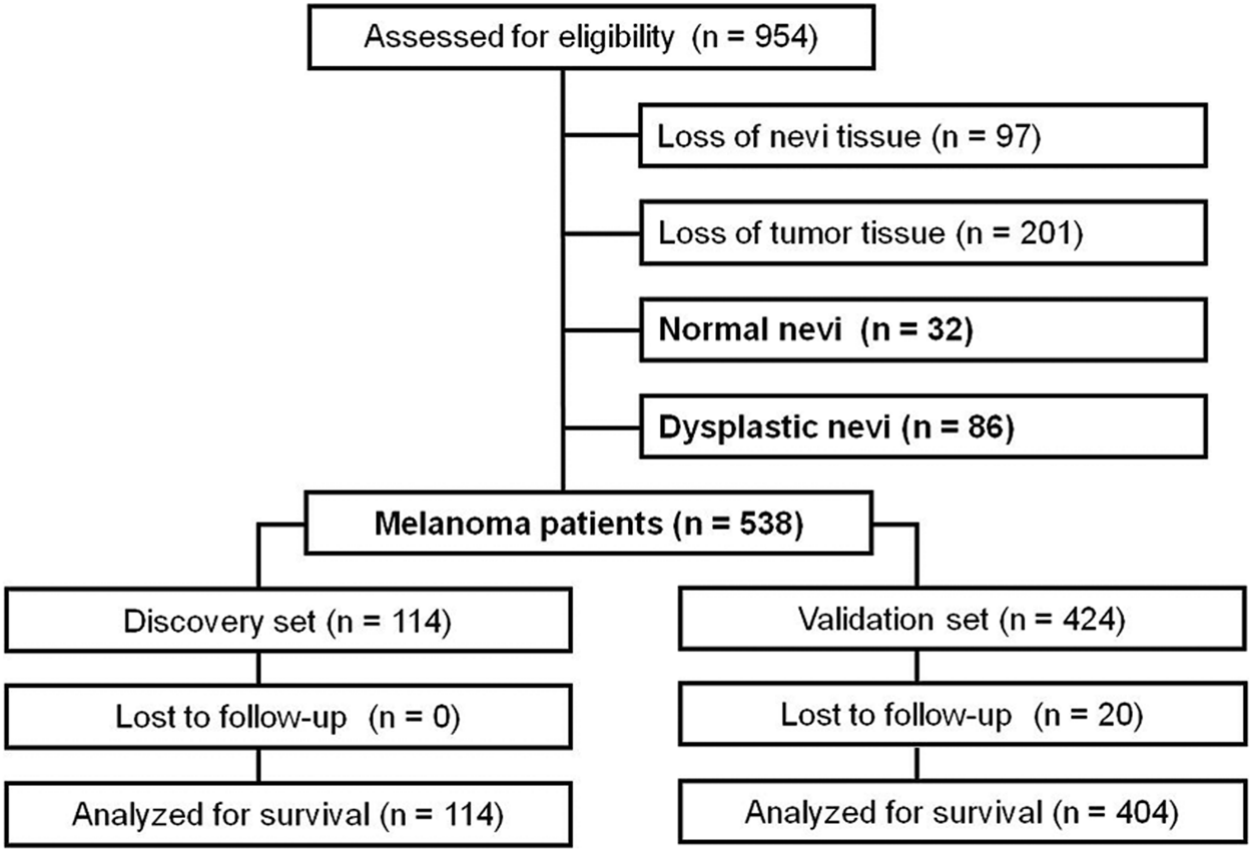
Diagram showing patient inclusion and exclusion.

Due to loss of biopsy cores or insufficient tumor cells present in the cores, 656 biopsies (32 normal nevi, 86 dysplastic nevi, 347 primary melanomas, and 191 metastatic melanomas) could be evaluated for Pin1 staining (Fig. 1). The survival status for 20 patients of this set was lost for follow-up; therefore, only 518 melanomas were subjected to followed survival analysis. The distribution of selected major clinical characteristics of melanoma patients in both discovery and validation sets are showed in Table 1.

**Table 1:** Clinicopathologic Characteristics of Melanoma Patients.

Variables	Discovery Set, No. (%)	Validation Set, No. (%)	Total, No. (%)
**Primary melanoma**			
Age, y			
≤60	31 (45.6)	140 (50.2)	171 (49.3)
>60	37 (54.4)	139 (49.8)	176 (50.7)
Sex			
Male	41 (60.3)	154 (55.2)	195 (56.2)
Female	27 (39.7)	125 (44.8)	152 (43.8)
Tumor thickness, mm			
≤1.0	17 (25.0)	102 (36.6)	119 (34.3)
1.01–2.00	23 (33.8)	70 (25.1)	93 (26.8)
2.01–4.00	11 (16.2)	50 (17.9)	61 (17.6)
>4.00	17 (25.0)	57 (20.4)	74 (21.3)
Ulceration			
Absent	53 (77.9)	228 (81.7)	281 (81.0)
Present	15 (22.1)	51 (18.3)	66 (19.0)
Subtype			
Lentigomaligna	14 (20.6)	59 (21.1)	73 (21.0)
Superficial spreading	27 (39.7)	104 (37.3)	131 (37.8)
Nodular	8 (11.8)	44 (15.8)	52 (15.0)
Acrolentigous melanoma	2 (2.9)	9 (3.2)	11 (3.2)
Unspecified	17 (25.0)	63 (22.6)	80 (23.0)
Site^a^			
Sun-protected	52 (76.5)	199(71.3)	251 (72.3)
Sun-exposed	16 (23.5)	80 (28.7)	96 (27.7)
**Metastatic melanoma**			
Age, y			
≤59	24 (52.2)	77 (53.1)	101 (52.9)
>59	22 (47.8)	68 (46.9)	90 (47.1)
Sex			
Male	33 (71.7)	101 (69.7)	134 (70.2)
Female	13 (28.3)	44 (30.3)	57 (29.8)
AJCC stage			
I	33 (29.0)	163 (38.4)	196 (36.4)
II	35 (30.7)	116 (27.4)	151 (28.1)
III	21 (18.4)	57 (13.4)	78 (14.5)
IV	25 (21.9)	88 (20.8)	113 (21.0)

AJCC indicates American Joint Committee on Cancer.

^a^Sun-protected sites: trunk, arm, leg, back, and feet; sun-exposed sites: head and neck.

No significant differences of were observed in the distribution of the age, sex, tumor thickness, ulceration, subtype, location and American Joint Committee on Cancer (AJCC) stage between the patients in the discovery and validation sets. Therefore, to increase statistical power, we combined the two sets. The total number of 347 cases of primary melanoma with ages ranging from 7 to 93 (median 60) was split into 195 male and 152 female cases (Table 1). Breslow thickness and AJCC criteria were used for melanoma staging. 212 cases of primary melanomas were less than 2.0 mm thick while 135 were thicker than 2.0 mm. 66 cases of primary melanoma showed ulceration, while 281 showed no signs of ulceration at diagnosis. Out of 191 metastatic melanomas (median age 59), 134 were male and 57 were female. In addition, 196 tumors were at AJCC stage I, 151 at stage II, 78 at stage III, and 113 at stage IV.

### Pin1 Expression is increased in Melanoma Cell Lines

**Figure 2:**
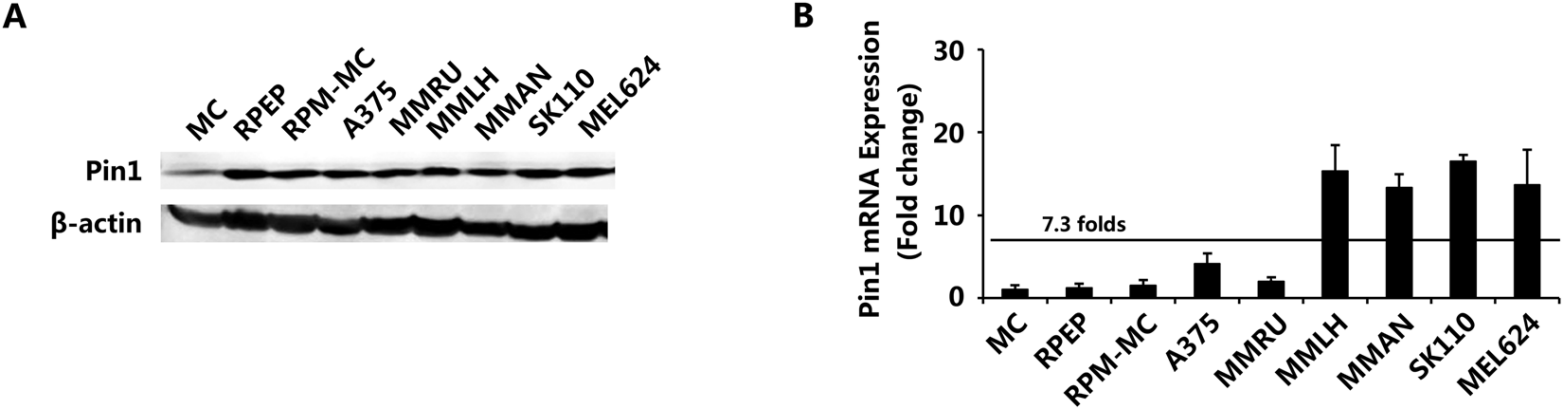
Pin1 expression in melanoma cell lines. Protein expression of Pin1 in melanoma cell lines as determined by Western Blot; (**B**) mRNA expression of Pin1 in melanoma cell lines.

We first investigated the expression level of Pin1 in melanoma cell lines and normal melanocytes by Western blot. Cell lines tested included the primary melanoma cell lines RPEP and RPM-MC, and the metastatic melanoma cell lines A375, MMRU, MMLH, MMAN, SK110 and MEL624. All melanoma cell lines (8/8) showed increased Pin1 protein levels compared with normal melanocytes (Fig. 2A). Pin1 mRNA as determined by RT-PCR was increased 7.3 folds on average in all melanoma cell linesas compared with normal melanocytes with 4/8 cell lines showing a >10-fold increase (Fig. 2B).

### Increased Cytoplasmic Pin1 Expression Correlates with Melanoma Progression

**Figure 3:**
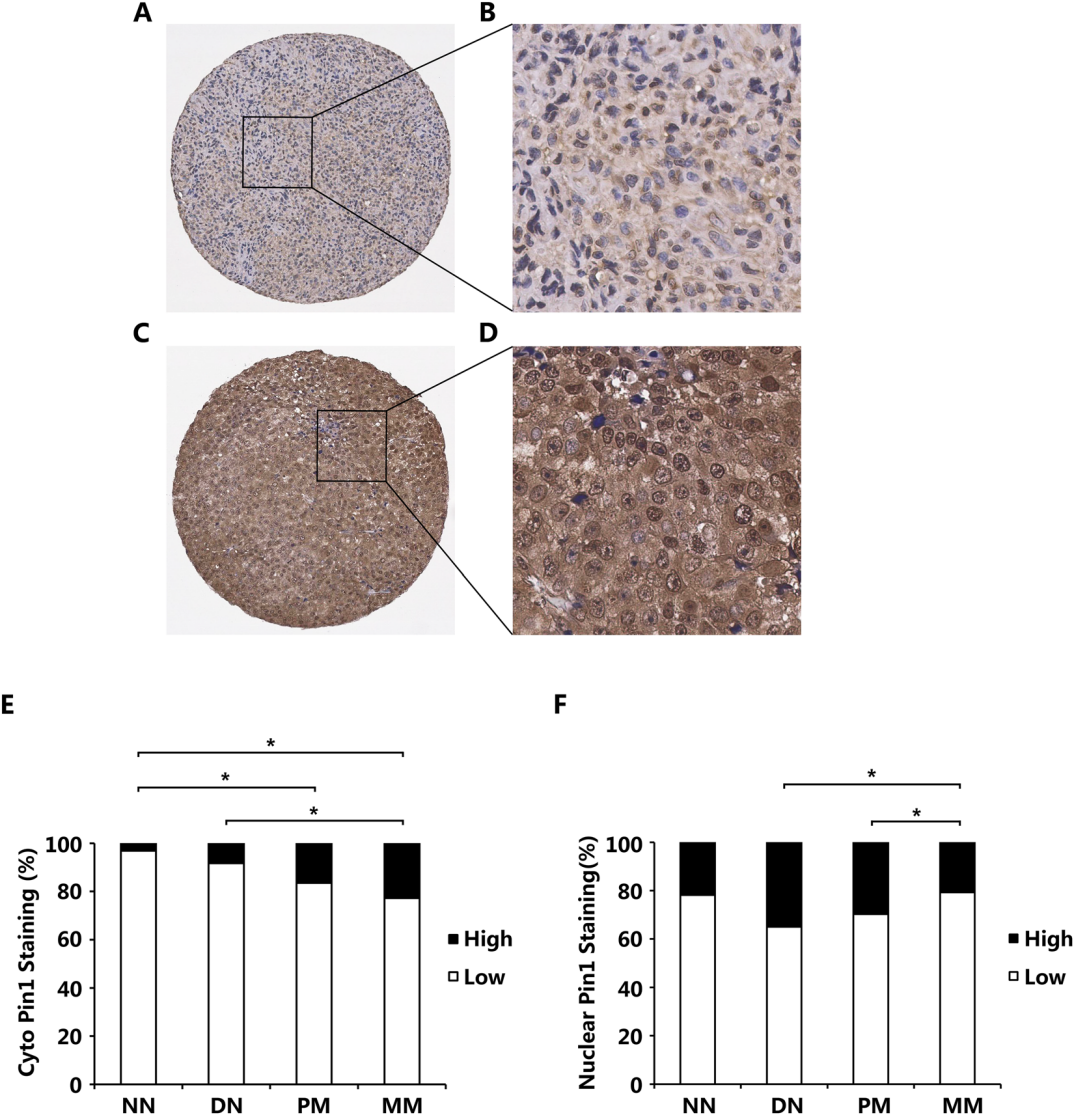
Correlation between Pin1 expression and melanoma progression. (**A**,**B**) Representative images showing low Pin1 immunohistochemistry staining. (**A**) Magnification: x10, (**B**). Magnification: x40; (**C**,**D**) Representative images showing high Pin1 immunohistochemistry staining. (**C**) Magnification: x10, (**D**). Magnification: x40; (**E**) Increased cytoplasmic Pin1 expression correlates with melanoma progression; (**F**) nuclear Pin1 expression first increased in DN, and decreased in PM and further decreased in MM. NN: normal nevi; DN: dysplastic nevi; PM: primary melanoma; MM: metastatic melanoma.

To further investigate the expression profiles of Pin1 in melanoma tissue, we conducted immunohistochemistry staining on both discovery set and validation set melanoma TMAs. According to X-tile software, we divided Pin1 staining into two categories: low (IRS: 0–8) and high (IRS: 9–12) (representative images are shown in Fig. 3A–D). The staining of Pin1 is present in both cytosol and nucleus and exhibits different distribution; hence cytoplasmic and nuclear Pin1 staining was evaluated separately. In the discovery set TMA, no significant difference in Pin1 expression was observed (Supplementary Fig. S1). In the validation set TMA, the fraction of cells with high cytoplasmic Pin1 expression was increased in primary and metastatic melanoma compared with nevi (*P* = 0.046 and 0.011, respectively, χ^2^ test) (Fig. 3E). The *P* value for the measured increase in cytoplasmic Pin1 expression in metastatic melanomas relative to dysplastic nevi was also very low (*P* = 0.016, χ^2^ test). However, nuclear Pin1 staining was increased when comparing normal nevi to dysplastic nevi (*P = *0.193, χ^2^ test) but decreased in a comparison of dysplastic nevi with primary melanoma (*P* = 0.031, χ^2^ test). A further decrease was noted in metastatic malignancies (*P* = 0.045, χ^2^ test).

### Pin1 Expression in Melanoma and Clinicopathologic Characteristics

In samples from all 538 melanoma patients, we investigated the correlation between cytoplasmic and nuclear Pin1 expression and clinicopathologic parameters. For cytoplasmic Pin1 expression, we did not find any significant differences between cytoplasmic Pin1 and clinical parameters (Table 2). For nuclear Pin1 expression, we found high nuclear Pin1 expression significantly decreased from 36% in stage I to 21% in stage II (*P* = 0.005, χ^2^ test) and further decreased to 9% in stage III (*P* = 0.000, χ^2^ test), but increased to 28% in stage IV (*P* = 0.004, χ^2^ test) (Supplementary Fig. S2). The mechanism underlying this phenomenon is unclear yet. Nuclear Pin1 expression decreased from thin melanoma (thinner than 2 mm) to thick melanoma (greater than 2 mm) (*P* = 0.001, χ^2^ test) (Supplementary Fig. S2). We did not find significant correlations between nuclear Pin1 expression and other clinicopathologic variables in primary melanoma. In addition, nuclear Pin1 expression was not correlated with age or sex of metastatic melanoma patients (Table 3).

**Table 2:** Cytoplasmic-Pin1 Staining and Clinicopathologic Characteristics of Melanoma Patients.

Variables	Cyto-Pin 1 Staining			
	Low, No. (%)	High, No. (%)	Total	P^a^
**Primary melanoma**				
Age, y				
≤60	152 (85.9)	25 (14.1)	177	0.872
>60	147 (86.5)	23 (13.5)	170	
Sex				
Male	169 (86.7)	26 (13.3)	195	0.760
Female	130 (85.5)	22 (14.5)	152	
Tumor thickness, mm				
≤1.0	104 (87.4)	15 (12.6)	119	0.855
1.01–2.00	79 (84.9)	14 (15.1)	93	
2.01–4.00	51 (83.6)	10 (16.4)	61	
>4.00	65 (87.8)	9 (12.2)	74	
Ulceration				
Absent	243 (86.5)	38 (13.5)	281	0.730
Present	56 (84.8)	10 (15.2)	66	
Subtype				
Lentigomaligna	69 (94.5)	4 (5.5)	73	0.231
Superficial spreading	110 (84.0)	21 (16.0)	131	
Nodular	43 (82.7)	9 (17.3)	52	
Acrolentigous melanoma	9 (81.8)	2 (18.2)	11	
Unspecified	68 (85.0)	12 (15.0)	80	
Site^b^				
Sun-protected	212 (76.5)	39 (71.3)	251	0.137
Sun-exposed	87 (23.5)	9 (28.7)	96	
**Metastatic melanoma**				
Age, y				
≤59	82 (82.0)	18 (18.0)	100	0.753
>59	73 (80.2)	18 (19.8)	91	
Sex				
Male	109 (81.3)	25 (18.7)	134	0.916
Female	46 (80.7)	11 (19.3)	57	
AJCC stage				
I	168 (85.7)	28 (14.3)	196	0.408
II	131 (86.8)	20 (13.2)	151	
III	65 (83.3)	13 (16.7)	78	
IV	90 (79.6)	23 (20.4)	113	

AJCC indicates American Joint Committee on Cancer.

^a^Chi - square test.

^b^Sun-protected sites: trunk, arm, leg, back, and feet; sun-exposed sites: head and neck.

**Table 3:** Nulcear-Pin1 Staining and or Characteristics of MelanomaPatients.

Variables	Nulcear-Pin 1 Staining			
	Low, No. (%)	High, No. (%)	Total	*P*a
**Primary melanoma**				
Age, y				
≤60	129 (72.9)	48 (27.1)	177	0.608
>60	128 (75.3)	42 (24.7)	170	
Sex				
Male	146 (74.9)	49 (25.1)	195	0.698
Female	111 (73.0)	41 (27.0)	152	
Tumor thickness, mm				
≤1.0	87 (73.1)	32 (26.9)	119	0.008
1.01–2.00	59 (63.4)	34 (36.6)	93	
2.01–4.00	47 (77.0)	14(23.0)	61	
>4.00	64 (86.5)	10 (13.5)	74	
Ulceration				
Absent	206 (73.3)	75 (26.7)	281	0.509
Present	51 (77.3)	15 (22.7)	66	
Subtype				
Lentigomaligna	56 (76.7)	17 (23.3)	73	0.977
Superficial spreading	95 (72.5)	36 (27.5)	131	
Nodular	39 (75.0)	13 (25.0)	52	
Acrolentigous melanoma	8 (72.7)	3 (27.3)	11	
Unspecified	59 (73.8)	21 (26.3)	80	
Site^b^				
Sun-protected	184 (73.4)	67 (25.7)	251	0.603
Sun-exposed	73 (74.7)	23 (25.3)	96	
**Metastatic melanoma**				
Age, y				
≤59	85 (85.0)	15 (15.0)	100	0.383
>59	73 (80.2)	18 (19.8)	91	
Sex				
Male	112 (83.6)	22 (16.4)	134	0.630
Female	46 (80.7)	11 (19.3)	57	
AJCC stage				
I	135 (68.9)	61 (31.1)	196	0.0001
II	122 (80.8)	29 (19.2)	151	
III	73 (93.6)	5 (6.4)	78	
IV	85 (75.2)	28 (24.8)	113	

AJCC indicates American Joint Committee on Cancer.

^a^Chi - square test.

^b^Sun-protected sites: trunk, arm, leg, back, and feet; sun-exposed sites: head and neck.

### Cytoplasmic Pin1 Expression is correlated with Melanoma 5-year Survival in the Discovery Set TMA

**Figure 4:**
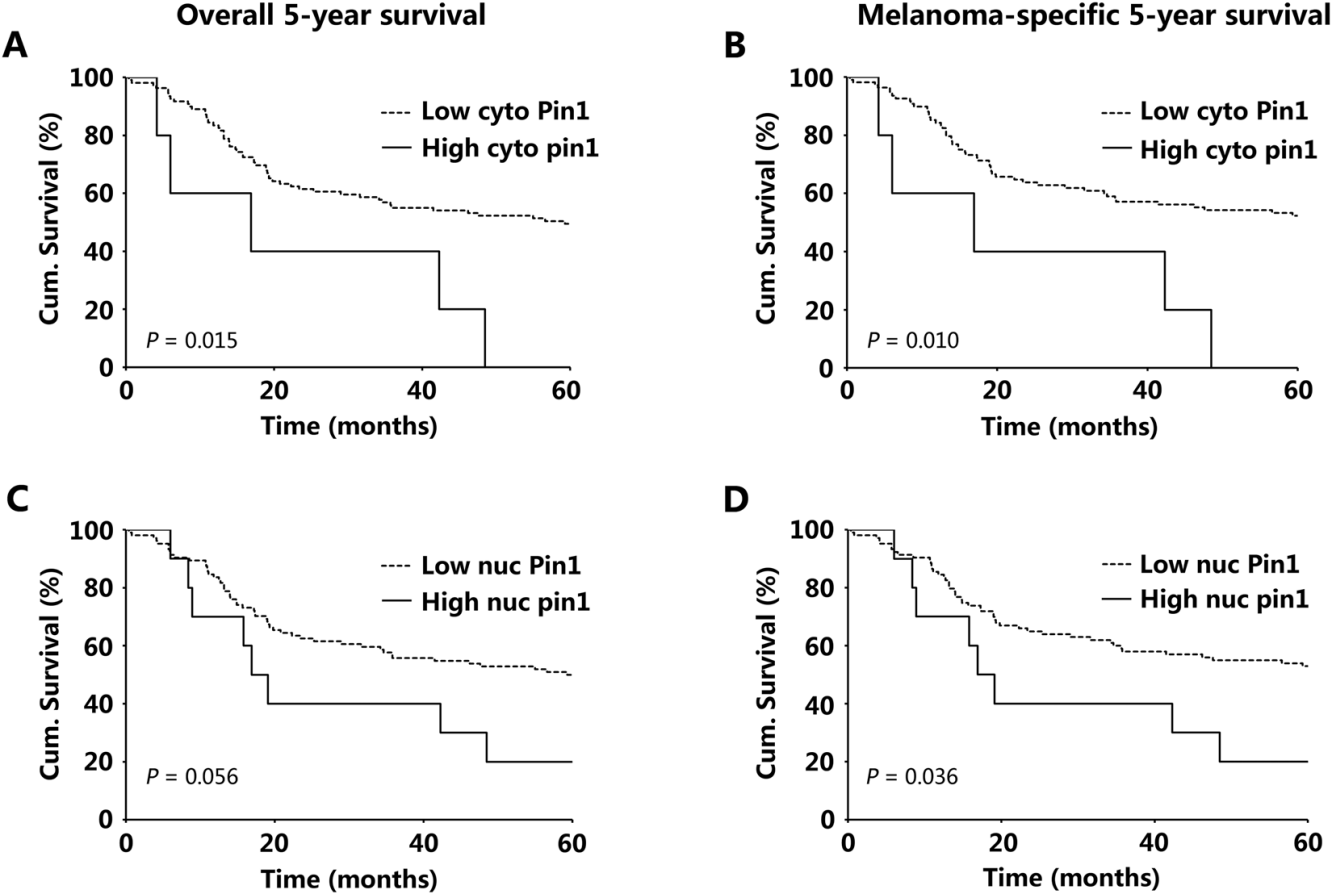
Kaplan-Meier survival analysis in discovery set TMA (114 cases) for cytoplasmic (**A**,**B**) and nuclear (**C**,**D**) Pin1 expression. Labels at the top of the figure apply to all graphs in the same column. Cum. Indicates cumulative.

To investigate the correlation between Pin1 expression and patient clinical outcome, we conducted Kaplan-Meier survival analysis on the discovery set tissue microarray. High expression of cytoplasmic Pin1 was correlated with worse patient 5-year overall survival and melanoma-specific survival (*P* = 0.015 and 0.010, χ^2^ test) (Fig. 4A,B). However, nuclear Pin1 expression was not significantly associated with melanoma-specific 5-year survival (Fig. 4C,D).

### Cytoplasmic Pin1 Expression is Correlated with Melanoma Patient 5-Year Survival in Validation Set TMA

**Figure 5:**
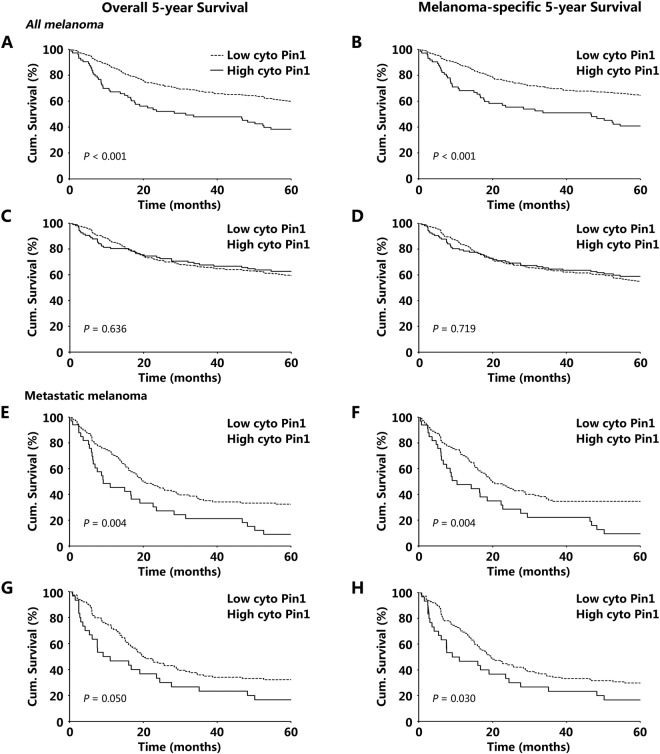
Kaplan-Meier survival analysis of all melanoma and metastatic melanoma patient in validation set TMA (404 cases) for cytoplasmic (**A**,**B**,**E**,**F**) and nuclear (**C**,**D**,**G**,**H**) Pin1 expression.

In validation set, a total of 404 samples of the TMA had complete clinical information. To further investigate the prognostic value of cytoplasmic/nuclear Pin1 expression, we constructed Kaplan-Meier survival analysis. Our data showed that overall 5-year survival in the high cytoplasmic Pin1 staining cohort was 38.07% compared to 64.96% in the low cytoplasmic Pin1 expression cohort. Statistical analysis revealed that the differences between high and low Pin1 expression cohorts are significant (overall survival, *P < *0.001; melanoma-specific survival, *P < *0.001) (Fig. 5). This data indicate that cytoplasmic Pin1 expression may serve as a promising prognostic marker in melanoma. However, similar to the discovery set, nuclear Pin1 expression was correlated neither with overall 5-year survival, nor with melanoma-specific 5-year survival (*P* = 0.636 and 0.719, respectively, log-rank test).

Furthermore, we investigated the correlation between Pin1 expression and patient survival in both primary and metastatic melanomas. In primary melanoma, only cytoplasmic Pin1 expression was associated with worse 5-year melanoma-specific survival (*P* = 0.035, log-rank test) (Supplementary Fig. S3).

Similarly, in metastatic melanoma, cytoplasmic Pin1 expression was associated with both overall and melanoma-specific 5-year survival (*P* = 0.004 and 0.050, respectively, log-rank test) (Fig. 5E,F). In contrast, nuclear Pin1 expression was only associated with melanoma-specific 5-year survival (*P* = 0.030, log-rank test) (Fig. 5H).

### Cytoplasmic Pin1 expression is an independent factor to predict melanoma patient survival

Finally, we conducted Multivariate Cox regression analysis to investigate the correlation between cytoplasmic Pin1 expression and melanoma patient survival. Our data showed that cytoplasmic Pin1 is an independent factor for predicting both overall and melanoma-specific patient survival (*P = *0.001 and 0.000, respectively, Table 4). Moreover, cytoplasmic Pin1 expression is also an independent factor for primary melanoma patient 5-year melanoma-specific survival (*P* = 0.039) (Table 4). Not surprisingly, we identified tumor thickness and ulceration status as the two most significant factors for predicting melanoma patient outcome (*P* = 0.001 and 0.003, respectively).

**Table 4:** Cox proportional regression analysis on 5-year overall and disease-specific survival of melanoma patients.

Variable^a^	Overall survival				Disease-specific survival			
	Univariate		Mutivariate		Univariate		Mutivariate	
	HR (95% CI)	*P*-value	HR (95% CI)	*P*-value	HR (95% CI)	*P*-value	HR (95% CI)	*P*-value
***All melanoma (n*** = ***403)***								
Age	0.795 (0.593–1.067)	0.127	0.710 (0.528–0.953)	**0.023**	0.873 (0.638–1.195)	0.398	0.765 (0.558–1.048)	0.095
Sex	0.936 (0.693–1.266)	0.670	1.268 (0.931–1.727)	0.133	0.976 (0.708–1.345)	0.881	1.380 (0.993–0.917)	0.055
AJCC	0.231 (0.171–0.313)	**0.000**	0.223 (0.163–0.304)	**0.000**	5.457 (3.915–7.605)	**0.000**	0.174 (0.124–0.245)	**0.000**
Cytoplasmic Pin1	0.515 (0.367–0.722)	**0.000**	0.525 (0.364–0.757)	**0.001**	2.097 (1.472–2.989)	**0.000**	0.499 (0.340–0.733)	**0.000**
Nuclear Pin1	1.086 (0.772–1.526)	0.637	0.849 (0.601–1.199)	0.352	0.936 (0.651–1.344)	0.719	1.025 (0.690–1.522)	0.903
***Primary melanoma (n*** = ***259)***								
Age	0.406 (0.248–0.665)	**0.000**	0.579 (0.343–0.976)	**0.040**	0.428 (0.244–0.753)	**0.003**	0.639 (0.352–1.160)	0.141
Sex	1.128 (0.713–1.786)	0.606	1.106 (0.692–1.768)	0.674	1.270 (0.749–2.155)	0.375	1.228 (0.714–2.111)	0.458
Thickness	0.281 (0.165–0.479)	**0.000**	0.418 (0.231–0.755)	**0.004**	0.191 (0.096–0.379)	**0.000**	0.292 (0.138–0.618)	**0.001**
Ulceration	0.271 (0.169–0.435)	**0.000**	0.459 (0.269–0.783)	**0.004**	0.217 (0.127–0.371)	**0.000**	0.406 (0.223–0.739)	**0.003**
Location	1.238 (0.746–2.055)	0.409	0.805 (0.481–1.347)	0.409	1.166 (0.615–2.212)	0.638	1.129 (0.590–2.158)	0.715
Cytoplasmic Pin1	1.613 (0.914–2.847)	0.099	0.555 (0.299–1.029)	0.062	1.929 (1.035–3.594)	**0.039**	0.474 (0.240–0.939)	**0.032**
Nuclear Pin1	1.202 (0.713–2.028)	0.490	1.014 (0.571–1.802)	0.961	1.238 (0.675–2.272)	0.490	1.033 (0.527–2.025)	0.925
***Metastatic melanoma (n*** = ***144)***								
Age	1.062 (0.724–1.560)	0.757	1.043 (0.703–1.547)	0.834	1.070 (0.723–1.583)	0.736	1.055 (0.706–1.578)	0.793
Sex	1.088 (0.723–1.638)	0.685	1.281 (0.847–1.939)	0.241	1.153 (0.763–1.743)	0.498	1.167 (0.884–2.042)	0.166
Cytoplasmic Pin1	0.743 (0.487–1.135)	0.170	0.571 (0.362–0.903)	**0.017**	0.738 (0.480–1.136)	0.167	0.760 (0.364–0.923)	**0.022**
Nuclear Pin1	0.842 (0.537–1.319)	0.453	0.794 (0.488–1.294)	0.355	0.800 (0.509–1.257)	0.333	0.921 (0.463–1.238)	0.267

HR, hazard ratio; CI, confidence interval; AJCC, American Joint Committee on Cancer.Bold indicates significant P values.

^a^Coding of variables: age was coded based on median age in patient cohorts: 1 (≦59 years) or 2 (>59 years) for all melanoma and primary melanoma patients, and 1 (≦60 years) or 2 (>60 years) for metastatic melanoma patients; Pin1 was coded as 1 (low) or 2 (high); thickness was coded as 1 (≦2 mm) or 2 (>2 mm); ulceration at the time of diagnosis was coded as 1 (no ulceration) or 2 (ulceration); location of lesions was coded as 1 (sun-protected area) or 2 (sun-exposed area); AJCC stage was coded as 1 (stage I and II) or 2 (stage III and IV).

## Discussion

The abnormally elevated expression of Pin1 occurs in a majority of malignancies. In the present study, aiming at an improved understanding of the role of Pin1 in melanoma progression, we used large-scale TMAs and immunohistochemistry to investigate Pin1 expression in 655 cases of pigmented skin lesions. Our data demonstrated that cytoplasmic Pin1 expression significantly increases with melanoma progression, and nuclear Pin1 expression shows a decrease in metastatic melanoma compared to early stage skin lesions. Furthermore, cytoplasmic Pin1 expression is significantly correlated with 5-year survival in metastatic as well as in all melanoma patient cohorts, and cytoplasmic Pin1 is an independent prognostic factor for melanoma patient survival. To our knowledge, this is the first study to investigate Pin1’s expression and prognostic value in melanoma using large-scale TMA and immunohistochemistry technology.

To date, very few publications focused on the role of Pin1 in melanoma. Jin and *et al*. have shown that suppression of Pin1 by miRNA interference inhibits proliferation and invasion *in-vitro* of A375 melanoma cells and suppresses their tumorigenic potential in athymic mice. This first functional and mechanistic study in melanoma demonstrated that down-regulation of Pin1 impedes tumorigenesis through inhibition of phosphorylation of Akt, C-Jun N-terminal kinase and pro-matrix metalloproteinase 2 (MMP2)^23^. A more recent study conducted by Kruiswijk and *et al*. revealed that Pin1 inhibition impaired the activity of the transcription factor FOXM1 and suppressed BRAF-V600E mutated metastatic melanoma cell survival^24^. Cell-permeable Pin1-FOXM1-blocking peptides were shown to inhibit the proliferation of freshly isolated human metastatic melanoma cells *ex vivo* and in 3D cultured patient-derived melanoids^24^. Another study has identified the novel covalent Pin1 inhibitor, KPT-6566, which impairs Pin1-dependent cancer formation and metastasis, indicating that therapeutic strategy based on Pin1 inhibition is promising^25^. Consistent with these observations, our study revealed that cytoplasmic Pin1 was significantly increased in melanoma cell lines and in primary and metastatic melanoma tissue biopsies, findings that also support the notion that cytoplasmic Pin1 is a promising therapeutic target for melanoma.

Our results suggest that elevated Pin1 activity might be required for melanoma transformation and progression. Previous study by Rustighi *et al*. has revealed that Pin1 is a Notch1 target and Pin1/ Notch1 interaction influents Notch1 transcription and activation in breast cancer^26^. A later study demonstrated that high level of Pin1 expression could maintain Notch signalling, which is an important pathogenesis mechanism in melanoma, and is associated with worse prognosis^27^. However, the regulatory mechanisms underlying this significant increase of cytoplasmic Pin1 expression in melanoma are largely unknown. Pin1 expression can be regulated both transcriptionally and post-translationally. E2 transcription factor 1 (E2F1) and several signalling cascades, such as Her2 and H-Ras, regulate *Pin1* transcription^28^. Death-associated protein kinase 1 (DAPK1) suppresses Pin1 activity by phosphorylating Pin1 at S71 in the Pin1 catalytic site and inhibits Pin1’s nuclear localization^29^. Additionally, PIN1 was shown to be a direct target of two members of the Notch protein family, Notch1 and Notch4^18,30^. Conversely, mixed-lineage kinase 3 (MLK3) phosphorylates Pin1 to enhance its catalytic activity and nuclear localization^31^. Moreover, Pin1 stability can be altered by ubiquitylation and SUMOylation following phosphorylation in both PPIase and WW domains^32–34^. The exact mechanism of regulation of Pin1 expression and cellular localization in melanoma as well as their functional consequences remains to be established.

Our data demonstrated that cytoplasmic Pin1 expression was negatively correlated with melanoma patient 5-year survival in the discovery set TMA (114 cases), a finding that was confirmed in the validation set TMA (404 cases). Moreover, Multivariate Cox proportional regression analysis indicated that high cytoplasmic Pin1 expression was an independent prognostic factor for melanoma. Interestingly, cytoplasmic Pin1 expression was also significantly associated with poor survival of patients with metastatic melanoma. However, cytoplasmic Pin1 expression was not correlated with primary melanoma clinical outcome. These results suggest that cytoplasmic or nuclear Pin1 may exert distinct functions in specific stages of melanoma progression.

## Materials and Methods

### Ethics Statement

The usage of human skin tissue samples and the waivers of patient consent in present research were approved by both the Clinical Research Ethics Board of Xiamen University and the University of British Columbia. The present study was conducted in accordance with the Declaration of Helsinki guidelines.

### Patient Biopsies and TMA Construction

We assembled 247 formalin-fixed, paraffin-embedded melanoma and control skin lesion tissues from the 1990 to 1999 archives of the Department of Pathology, Vancouver General Hospital into a discovery set. To validate the findings from the discovery set, we assembled an additional array of 559 melanoma tissues and 148 skin lesion tissues collected between 1992 and 2009 as the validation set. Patients include in this cohort were prospectively followed up until death or the latest follow-up. During the follow-up period, 20 patients were lost to follow-up; 214 died of melanoma, while 33 died from other causes. Each TMA section (4 μm) was routinely stained with hematoxylin and eosin, as well as melanocyte marker S-100.

### Immunohistochemistry of TMAs

TMA slides were immunohistochemically stained as described previously^35^. The monoclonal mouse anti-Pin1 antibody (Cat # MAB2294) (1:50 dilution; R&D Systems, MN, USA) was diluted 1:100 and used. Negative controls were performed by omitting the Pin1 antibody during the primary antibody incubation.

### Evaluation of Immunostaining and Statistical Analysis

Blind evaluation of Pin1 staining was performed by a trained dermatopathologist and two observers joining through a multiple viewing microscope, and a consensus was reached for the score of each core. The Pin1 staining intensity was scored as 0, 1, 2 and 3, and the percentage of positive Pin1 staining cells was scored as 1 (1–25%), 2 (26–50%), 3 (51–75%) and 4 (76–100%). When duplicated cores show different staining, the higher score from the two tissue cores was taken as the immune reactivity score (IRS)^36^. The final score was calculated by multiplying the scores of staining intensity and the percentage of positive cells. Based on IRS, Pin1 staining pattern was defined as: low (IRS: 0–8) and high (IRS: 9–12). The optimal cut-off points were determined using the X-tile software (Yale University). Statistical analysis was conducted using the SPSS version 21 software (SPSS Inc, Chicago, IL).

### Quantification of Pin1 Expression Levels in Melanoma Cell Lines

Protein was extracted from cells as previously described^37^. Mouse anti-Pin1 antibody (MAB2294, R&D Systems, MN, USA) was used to detect the Pin1 protein expressio. β-actin was used as the internal reference (1:10000 dilution, sigma, Oakville, ON, Canada).

RNA was extracted from cells as described previously^37^. Pin1 expression was adjusted using the reference gene *GAPDH*.

Primer sequences:

Pin1-forward: TCGGGAGAGGAGGACTTTG

Pin1-reverse: GGAGGATGATGTGGATGCC

GAPDH-forward: AAGATCATCAGCAATGCCTCC

GAPDH-reverse: TGGACTGTGGTCATGAGTCCTT.

## Electronic supplementary material

Supplementary Informatiom
