# Supplementary material for: Cytoplasmic Pin1 expression is increased in human cutaneous melanoma and predicts poor prognosis
Source: Sci Rep. 2018 Nov 15;8:16867. doi: 10.1038/s41598-018-34906-6 (PMC6238011; doi:10.1038/s41598-018-34906-6)

**Supplementary Information**

**Cytoplasmic Pin1 expression is increased in human cutaneous melanoma and predicts poor prognosis**

Xin Chen^1,2,*^, Xiaosong Liu^3,*^, Bin Deng^4^, Magdalena Martinka^5^, Youwen Zhou^6^, Xiaopeng Lan ^1^ and Yabin Cheng^3^.

**Supplementary Figure S1**. In discovery set TMA, both cyto- and nuc- Pin1 are not significantly associated with melanoma progression. DN, dysplastic nevi; PM, primary melanoma; MM, metastatic melanoma.


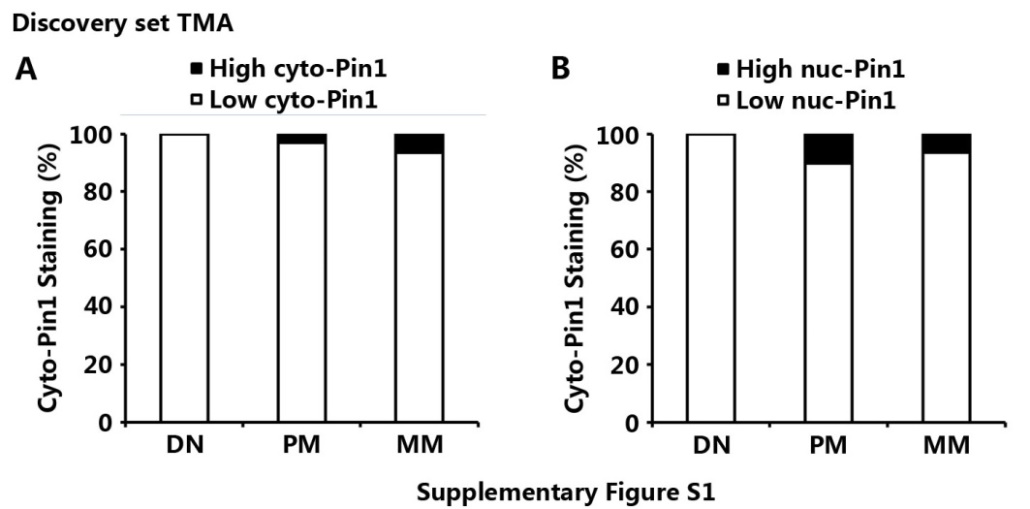


**Supplementary Figure S2**. In validation set TMA, correlation between cyto- and nuc-Pin1 and tumor thickness and AJCC stages. (**A**) No significant difference of cyto-Pin1 expression between thin (< 2 mm) and thick tumor (≥ 2 mm) (*P* = 0.906, Chi-square test); (**B**) Tumors ≥ 2 mm thick have a high percentage of high nuc-Pin1 expression compared with tumors less than 2 mm (*P* = 0.001, Chi-square test); (**C**) No significant differences of cyto-Pin1 expression across four AJCC stages; (**D**) Tumors in AJCC stage II and III have a low percentage of high nuc-Pin1 expression compared with stage I (*P* = 0.005 and < 0.001, respectively, Chi-square test), tumors in stage IV have a high percentage of high nuc-Pin1 expression compared with stage III (P = 0.004, Chi-square test)


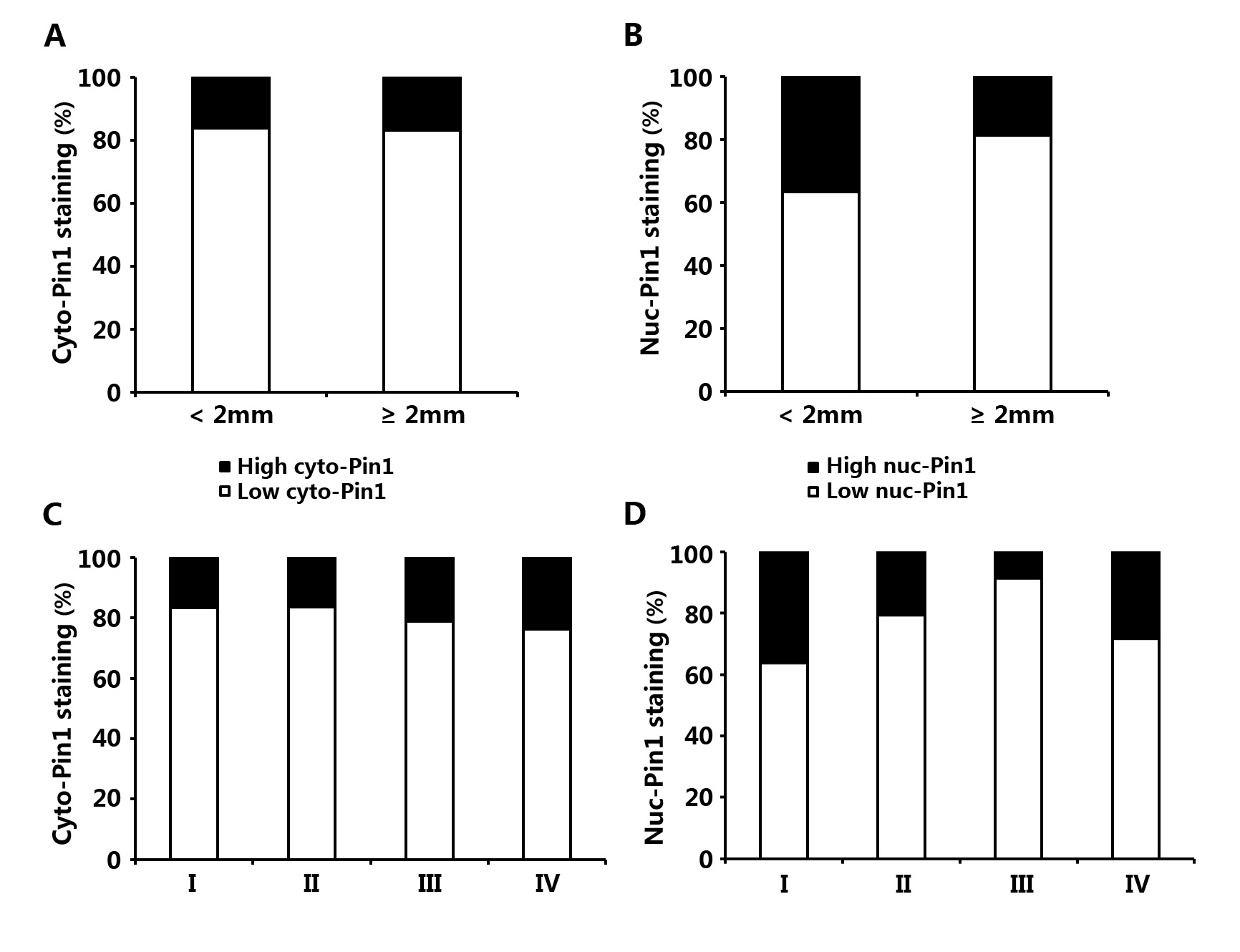


**Supplementary Figure S3**. Kaplan-Meier survival analysis of primary melanoma patients in validation set TMA. (**A, B**) Patients with high cyto-Pin1 expression have a significantly worse melanoma-specific 5-year survival (*P* = 0.035, log-rank test), but not significant in overall 5-year survival (*P* = 0.096, log-rank test). (**C, D**) nuc-Pin1 expression are not significantly associated with both overall and melanoma-specific 5-year survival (*P* = 0.489 and 0.489, respectively, log-rank test).


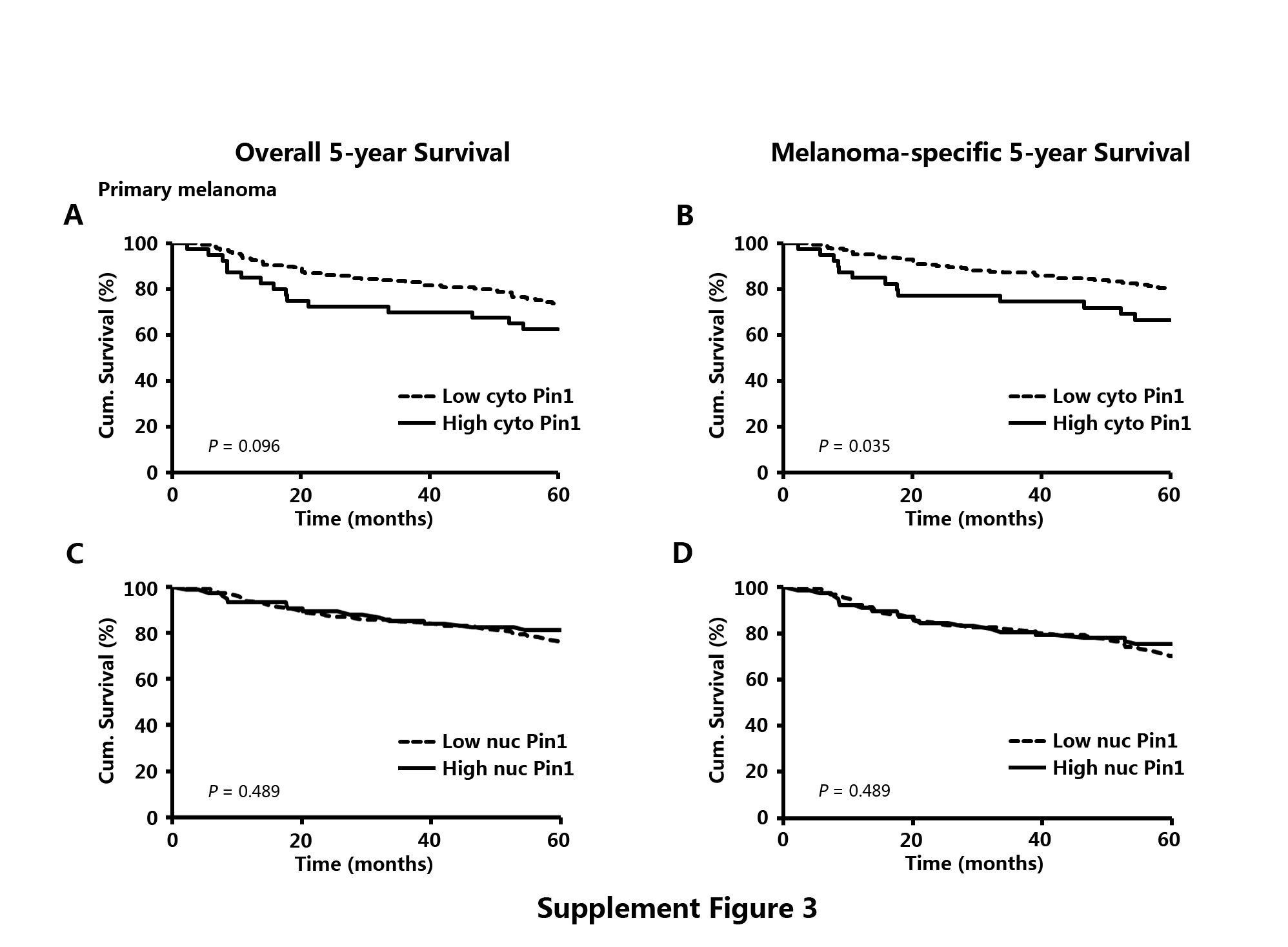


**Supplementary Figure S4**. (**A**) Protein expression of Pin1 in melanoma cell lines as determined by Western Blot. (**B**) Protein expression of β-actin in melanoma cell lines as determined by Western Blot.


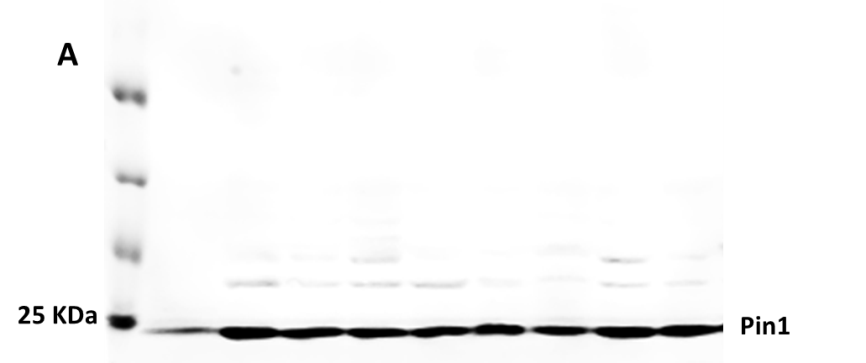


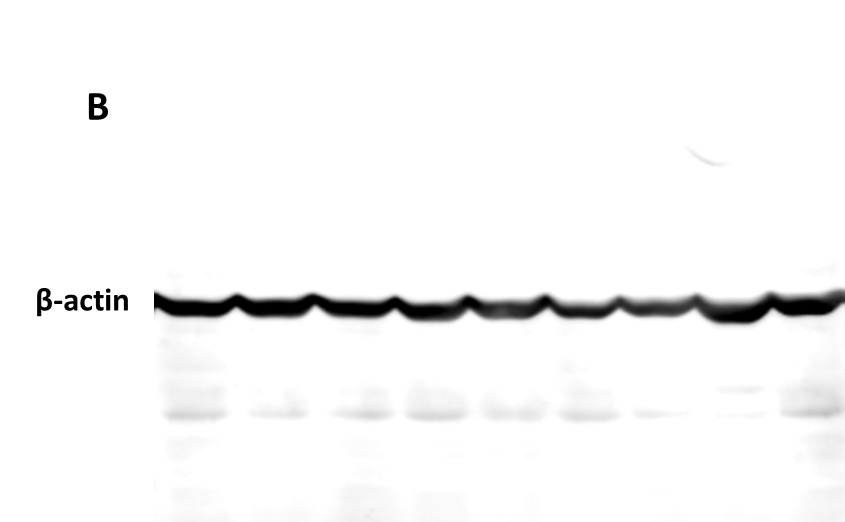

Supplement: Supplementary file 1 — Supplementary Informatiom [file 41598_2018_34906_MOESM1_ESM.docx]
